# Effect of WC/Co coherency phase boundaries on Fracture toughness of the nanocrystalline cemented carbides

**DOI:** 10.1038/srep31047

**Published:** 2016-08-03

**Authors:** Hongxian Xie, Xiaoyan Song, Fuxing Yin, Yongguang Zhang

**Affiliations:** 1 School of Mechanical Engineering, Hebei University of Technology, Tianjin 300132, China; 2 College of Materials Science and Engineering, Key Laboratory of Advanced Functional Materials, Education Ministry of China, Beijing University of Technology, Beijing 100124, China; 3 Tianjin Key Laboratory of Materials Laminating Fabrication and Interface control Technology, Hebei University of Technology, Tianjin 300132, China; 4 Research Institute for Energy Equipment Materials, Hebei University of Technology, Tianjin 300132, China

## Abstract

The effect of coherency WC/Co phase boundaries on the fracture toughness of the nanocrystalline WC-Co cemented carbides is studied by MD simulation method. The simulation results show that the nanocrystalline WC-Co cemented carbides with coherency WC/Co phase boundaries has higher fracture toughness than that without coherency WC/Co phase boundaries. Moreover, the mechanism of why coherency WC/Co phase boundaries can improve the fracture toughness of the nanocrystalline cemented carbides is also investigated. It is found the fact that the separation energy of the coherent WC/Co phase boundary is larger than that of the incoherent WC/Co phase boundaries is the main reason for this excellent mechanical property.

The WC-Co cemented carbides are widely used in industries owing to their excellent mechanical properties^1^,^2^,^3^. Examples of typical applications of the WC-Co cemented carbides are cutting, machining, mining, drilling tools and wear parts. Hardness and fracture toughness are the two most important mechanical properties of the WC-Co cemented carbides; other mechanical properties, such as impact resistance and wear resistance, are fundamentally dependent on the hardness and fracture toughness. As described by the Hall-Petch relation^4^,^5^, the hardness of the WC-Co cemented carbides increases with decreasing grain size. Therefore intensive efforts have been made to study the WC-Co cemented carbides with ultrafine or nanocrystalline grain structures^6^,^7^,^8^,^9^,^10^. However, it is well-known that the fracture toughness of the WC-Co cemented carbides is inversely proportional to the grain size; so a finer grain size usually results in lower fracture toughness^11^,^12^,^13^. Naturally, how synthesizing WC-Co cemented carbides with both high hardness and high fracture toughness is still challenging.

Recently, using nanoscale WC–Co composite powder and the spark plasma sintering technique, a WC-Co cemented carbide bulk with a mean grain size of about 65 nm was synthesized by our group^14^. This nanocrystalline WC-Co cemented carbide bulk possesses a superior combination of hardness and fracture toughness. By using Vickers hardness tester and indentation method, the hardness and fracture toughness were measured as *H*_*V*_ = 2050 ± 10 *kg fmm*^−2^ and 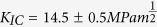, respectively; which is more excellent than that of other cemented carbides reported in the literature with the same Co content^15^,^16^,^17^,^18^,^19^. It is understandable that the high hardness of the present nanocrystalline WC-Co cemented carbide came from its smaller grain size; however, its high fracture toughness is not well-understand and need further investigation.

The microstructures of the present nanocrystalline WC-Co cemented carbide are examined by transmission electron microscopy (TEM) and high-resolution TEM, it is found that there are plenty of coherent WC(0001)/Co(111) phase boundaries in the randomly selected areas for TEM observations; these coherent WC/Co phase boundaries have been speculated as the main underlying reason for the high fracture toughness. However, in the literature there still lacks theoretic or experimental works to further investigate the deformation mechanism as well as the relationship between the coherent phase boundaries and the high fracture toughness of the nanocrystalline WC-Co cemented carbide. In the present work, we will study the deformation mechanism of the nanocrystalline WC-Co cemented carbide with and without coherent WC/Co phase boundary by using molecular dynamics (MD) simulation method, and discuss the relationship between the high fracture toughness and the coherent WC/Co phase boundaries.

Two types of nanocrystalline WC-Co cemented carbide model containing about 2.37 million atoms are generated from Voronoi construction (see methods). The analytical bond order potential (ABOP) for the W–C–Co system^20^,^21^ is adopted, which was developed using first principles calculations and experimental data. This type potential has been successfully applied to describe metallic and covalent bonding, as well as to a metal-carbide system^22^,^23^,^24^,^25^,^26^. All MD simulations are carried out with LAMMPS code^27^. MD simulations are conducted using isothermal-isobaric ensemble via Nose-Hoover thermostat^28^,^29^, and periodic boundary conditions are imposed in all three directions. The initial constructed models are relaxed at 300 K for 100 picoseconds (the time step is 2 femtosecond) to reach a stable structure. Then the models are deformed in uniaxial tension along x direction until failure at constant strain rates (5 × 10^8^*s*^−1^) with a stress-free condition for the other two directions. The stress is calculated using the virial definition without the kinetic portion^30^,^31^,^32^.

## Results

**Figure 1:**
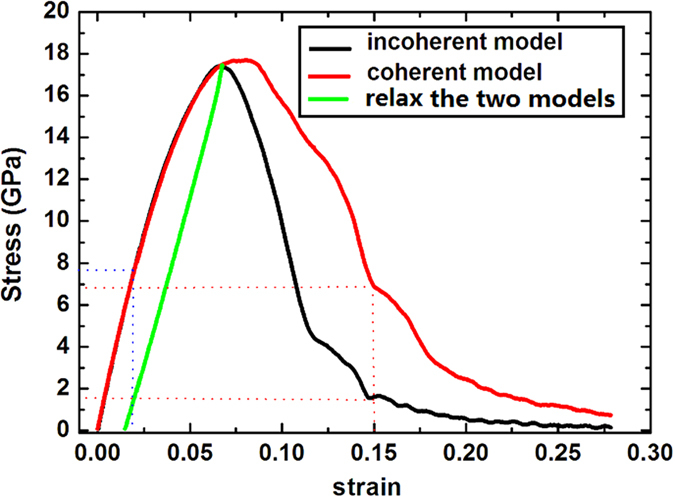
Stress-strain curves of tensile deformation applied to coherent and incoherent models.

Figure 1 illustrates stress-strain curves of the two models. As shown, below strain value of 6.6%, the two stress-strain curves are almost completely overlapped and the stress increase with the increasing of the strain, indicating that at the present stage no crack occurs and the deformation mechanism of the two models is insensitive to the joint types of the WC/Co phase boundaries. Moreover, the present stage can be further divided into two sub-stages: below strain value of 2.1%, the stress is directly proportional to strain, and the Young’s modulus holds constant. When the strain value is between 2.1% to 6.6%, nonlinear stress-strain behavior can be found, and the Young’s modulus decreases with the increasing of strain. The different stress-strain behaviors of the two sub-stages maybe suggest different deformation mechanisms of the two nanocrystalline WC-Co cemented carbide models. Beyond the strain value of 6.6%, the stress will drop, at the same time cracks begin to nucleate at the WC grain boundaries. From the figure it can be found that the stress-strain behavior of the two models is remarkably different: the stress of the coherent model drops more slowly than that of the incoherent model (for instance, at 15% strain the stress of the incoherent model drops to 1.6 GPa, however, the stress of the coherent model only drops to 6.9 GPa). This indicates that the coherent model possesses higher fracture toughness than the incoherent model.

Furthermore, the Fig. 1 also displays the relaxation process of the two models. If the two models are relaxed from the highest stress pointes of the two stress–strain curves, the stress linearly decreases with the decreasing of the strain. When the stress decreases to zero, the strain only decreases to 1.49%; this indicates that the two models have conducted a plastic deformation process during the tensile process.

**Figure 2:**
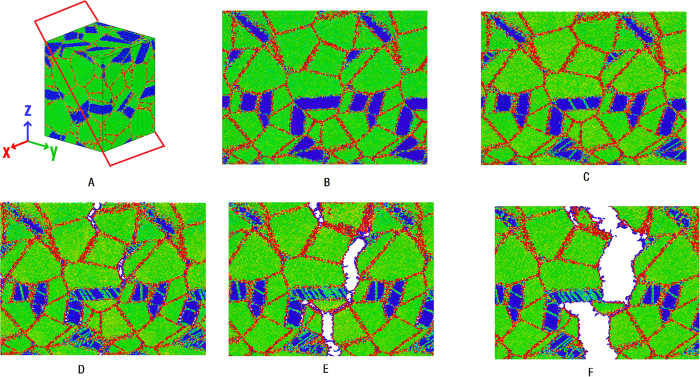
Cross-section views of the atomic configurations of the incoherent model during tensile deformation. Atoms are colored by theirs centro-symmetry parameter, red lines represent WC grain boundaries and incoherent WC/Co phase boundaries; green and blue areas represent WC and Co grains, respectively; green lines in Co grains represent stacking faults.

**Figure 3:**
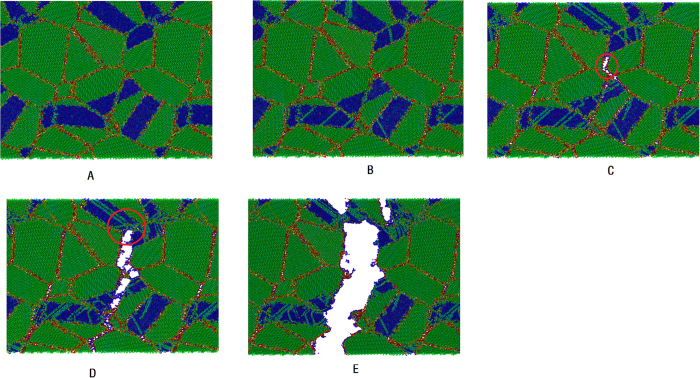
Cross-section views of atomic configurations of the coherent model during tensile deformation. Atoms are colored by theirs centro-symmetry parameter, red lines represent WC grain boundaries and incoherent WC/Co phase boundaries; green and blue areas represent WC and Co grains, respectively; green lines in Co grains represent stacking faults. WC/Co phase boundaries without red color are coherent phase boundaries.

In order to further understand the different stress-strain behaviors between the coherent and incoherent models, in this part we study the deformation mechanism of the two models. Figures 2 and 3 give the cross-section views of the incoherent and coherent models during tensile deformation. In the figures atoms are colored by theirs centro-symmetry parameter^33^. Red lines represent WC (or Co) grain boundaries and incoherent WC/Co phase boundaries; green and blue areas represent WC and Co grains, respectively; green lines in Co grains represent stacking faults; WC/Co phase boundaries without red color represent coherent phase boundaries.

By carefully observing the Fig. 2, we find that below strain value of 2.1%, the model is in the linear elastic stage, no dislocation is found. Beyond 2.1%, partial dislocations begin to nuclear at the Co grain boundaries and WC/Co phase boundaries, subsequently slip in the Co grains and leave stacking faults (SF) behind them (some SF are highlighted by red circle in Fig. 2C). This stage continues to strain value of 6.6%. The activation of dislocations in Co phase can release local stress at the Co grain boundaries and WC/Co phase boundaries, increase the ductility of the nanocrystalline WC-Co cemented carbide. However, because no dislocation activates in WC grains, there must be accumulated high local stress at the WC grain boundaries with the increasing of the strain. When strain value reaches 6.6%, cracks begin to nuclear at the triple junction region of the WC grain boundaries (some cracks are highlighted by red circle in Fig. 2D). When the model is further deformed from the strain value of 6.6% to 26.6%, the cracks will propagate along the grain boundaries or phase boundaries, then interconnect with each other and form a main crack, and the propagation of which finally leads to the fracture of the model.

From Fig. 3, we also find that below strain value of 2.1%, the coherent model is in the linear elastic stage, no dislocation can be found. Beyond the strain value of 2.1%, partial dislocations begin to nuclear at the Co grain boundaries and WC/Co phase boundaries, then slip in the Co grains and leave stacking faults (SF) behind them (some SF are highlighted by red circle in Fig. 3B). Below the strain value of 6.6%, the deformation mechanism of the coherent model is similar to that of the incoherent model, and this is the underlying reason why the stress-strain curves of the two different models are almost completely overlapped. Form the above analysis we can conclude that before cracks nuclear, the mechanical property of the nanocrystalline WC-Co cermet materials is insensitive to the joint types of the WC/Co phase boundaries. With accumulating high local stress at the WC grain boundaries, cracks begin to nuclear at the triple junction region of the WC grain boundaries at strain value of 6.6%, (some cracks are highlighted by red circle in Fig. 3C), When the model is further deformed from the strain of 6.6% to 26.6%, the cracks will propagate along the WC grain boundaries or incoherent WC/Co phase boundaries, then interconnect with each other and form a main crack. Noteworthily, when the main crack tip arrived at the coherent WC/Co phase boundary (the main crack tip is highlighted by red circle in Fig. 3D), the crack will propagate through the Co phase and form a transcrystaline path rather than along the coherent WC/Co phase boundary. Furthermore, the FCC Co in the present model is equipped with more sliding systems than HCP Co, the dislocations in FCC Co are easier to slide under the stress field of the crack tip, thus crack tip blunting can be achieved^34^,^35^,^36^; subsequently, Co ligament bridging can be formed at the crack tip (Co ligament bridging is highlighted by red circle in Fig. 3E). The high ductility of the FCC Co phase can increase the work of rupture, so increase the fracture toughness of the nanocrystalline WC-Co cemented carbides. To remove statistical uncertainty, other five pairs of incoherent and coherent samples have been further investigated, it is found that coherent WC/Co boundaries indeed increase the probability of transcrystalline path.

Synthetically considering the stress-strain behavior and the deformation mechanism of the two models, we find that the tensile process of the two models can be divided into three different stages: the linear elastic stage (strain value from 0% to 2.1%), plastic stage (from 2.1% to 6.6%) and fracture stage (larger than 6.6%). In the plastic stage, the plastic deformation only occurs in the FCC Co phase rather than WC phase, which indicates that only FCC Co phase can contribute to the plasticity of the two models. Moreover, the stress-strain curves of the two models are almost completely overlapped in the first two stages, and different from each other only in the fracture stage; suggesting that the WC/Co coherency phase boundaries only works in the fracture stage rather than in the first two stages.

## Discussion

**Figure 4:**
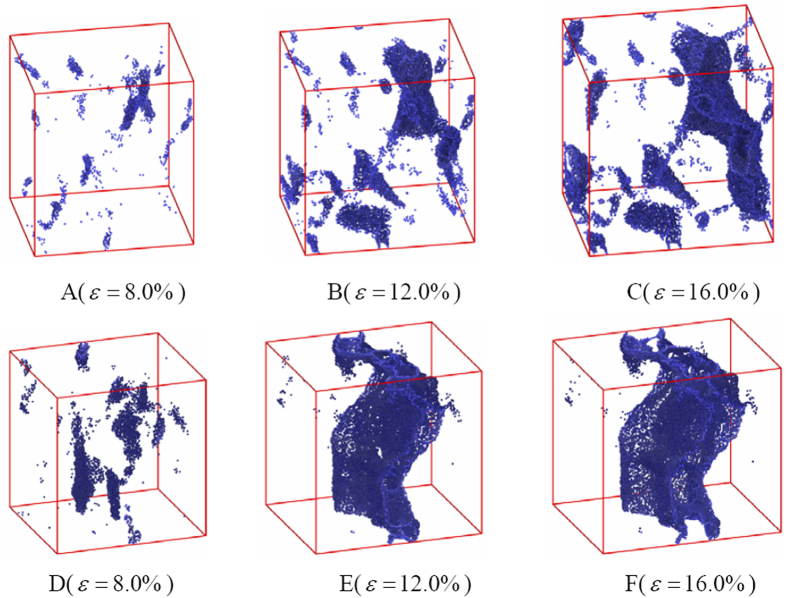
Evolution of total crack with tensile strain for coherent and incoherent models. (**A–C**) display the evolution of total crack for coherent model; while (**D,E**) and (**F**) display that of incoherent model.

To further study the effect of WC/Co coherency phase boundaries on the fracture process of the two models, the evolution of the total cracks in the two different models during the tensile process are displayed in Fig. 4. As periodic boundary condition is applied to the two models, atoms with large Voronoi volumes can be considered as the atoms located at cracks’ surface. The Voronoi volume of a perfect FCC Co and HCP WC atom are 11.08 Å^3^ and 10.17 Å^3^, respectively. In the present works, we use the average value (25.00 Å^3^) as a critical value to determine whether an atom is at crack surface; atoms with Voronoi volume higher than the critical value are identified as crack surface atoms. From the Fig. 4 it can be found that the microcracks in the two models are nucleated at multiple sites (Fig. 4A,D). For the coherent model, the coherent WC/Co phase boundary maybe block the propagation of microcracks along them, so increases the probability of transcrystaline path through the FCC Co phase. The high ductility of the FCC Co phase can increase the work of rupture and decreases the probability to form a main crack (Fig. 4B,C). However, for the incoherent model, because there are not coherent WC/Co phase boundaries, microcracks will propagate along the grain or phase boundaries, then interconnect with each other and finally form a main crack (Fig. 4E,F).

**Figure 5:**
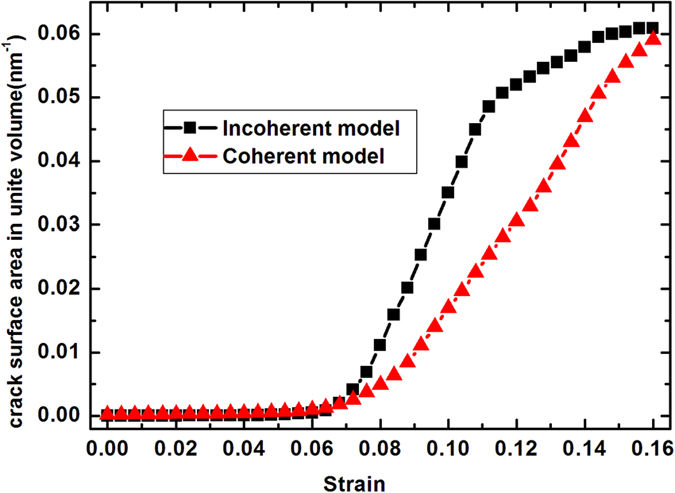
Evolution of crack fraction (crack surface/system volume) with tensile strain for incoherent and coherent models.

To quantitatively analyze the fracture process of the two models, the crack surface area per volume (i.e., the ratio of the crack surface area to the total volume of the system) is calculated during deformation. We suppose that each crack surface atom contribute 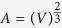 (*V* represents Voronoi volume of a FCC Co or HCP WC atom in perfect lattice) area to the crack surface, and the *A* of such atoms are then summed up as the total crack surface inside the model. The calculated crack surface area per volume as a function of strain for both coherent and incoherent models are shown in Fig. 5, which indicates that both of the two models begin to form cracks almost at the same strain value; and the crack surface area per volume in the coherent model is always smaller than that of the incoherent model at the same strain level. The figure suggests that the coherent WC/Co phase boundary is stronger than the incoherent one, and shows higher resistance to intergranular cracking. The difference in the crack surface area per volume between the coherent and incoherent models can well explain why the coherent model’s fracture toughness is higher than that of the incoherent model.

**Figure 6:**
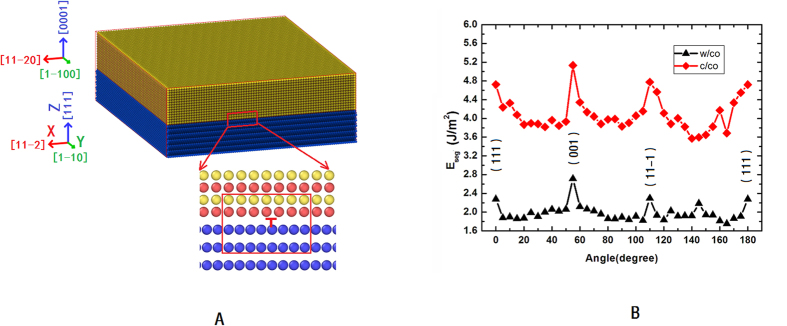
The model for calculating the separation energy of the WC/Co phase boundary and the separation energies for various WC/Co phase boundaries.

As we know, separation energy is usually used to assess the strength of grain and phase boundary^37^,^38^. To further study the effect of coherent WC/Co phase boundary on the fracture toughness of the nanocrystalline cemented carbides, we calculate and compare the separation energies for various WC/Co phase boundaries. In order to calculate the separation energy, we first construct a model with a coherent WC/Co phase boundary as Fig. 6A, relax it by the conjugate gradient method and record the system’s energy as *E*_*WC/Co*_; then separate the model from the WC/Co phase boundary, relax it and again record the system’s energy as *E*_*WC*+*Co*_. Finally, the separation energy of the coherent WC/Co phase boundary is calculated as:


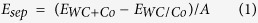


where *A* is the area of the WC/Co phase boundary. To construct the incoherent WC/Co phase boundary, we hold the WC part still and rotate the Co part by an angle about Y axes; then calculate the separation energies of the incoherent WC/Co phase boundary using the same method as above. Since the stoichiometric WC (0001) has two different surfaces: one C-terminated, and the other W-terminated; therefore there are two different types WC/Co phase boundaries. We name the C-terminated WC/Co phase boundary as C/Co type phase boundary and the W-terminated WC/Co phase boundary as W/Co type phase boundary.

The separation energies for various WC/Co phase boundaries are displayed in Fig. 6B, from which it can be found that except for WC(0001)/Co(001) phase boundary, the separation energy of the coherent WC(0001)/Co{111} phase boundary is larger than those of incoherent WC/Co phase boundaries, suggesting that the coherent WC(0001)/Co{111} phase boundary is much stronger than the incoherent WC/Co phase boundaries. Therefore, the existence of the coherent WC/Co phase boundary in the nanocrystalline cemented carbides will effectively block the propagation of microcracks along them, increases the probability of transcrystaline path through the FCC Co phase. The high ductility of the FCC Co phase can increase the fracture toughness of the nanocrystalline cemented carbides. Interestingly, we find that the separation energy of WC(0001)/Co(001) phase boundary is a little larger than that of the coherent WC(0001)/Co{111} phase boundary, indicating that if we manage to increase the probability of the WC(0001)/Co(001) phase boundary, we also can increase the fracture toughness of the nanocrystalline cemented carbides.

As we know, hardness (or strength) and fracture toughness are the two most important mechanical properties of the structural materials. Most engineering designs call for structural materials that possess both high hardness and high fracture toughness; however, hardening usually compromises the fracture toughness and vice versa^39^. Grain refinement is one of methods to harden materials because the grain boundaries can effectively obstruct the lattice dislocation motion. However, grain boundary is a much weak part of the material (has low separation energy), and beneficial to form intergranular crack. Therefore, a finer grain size usually results in lower fracture toughness^11^,^12^,^13^. Naturally, increasing the density of high separation energy grain boundaries is a promising way to obtain material with both high hardness (or strength) and high fracture toughness. Following this idea, nanotwinned Cu with both of high strength and high fracture toughness has been synthesized^40^,^41^; moreover, ultrahard nanotwinned cubic boron nitride and nanotwinned cubic diamond are also has been successfully obtained^42^,^43^. All these outstanding works use twin boundary, which possesses high separation energy, to realize both high hardness (or strength) and high fracture toughness. Our group use coherent WC(0001)/Co{111} phase boundary, which also possesses high separation energy, to successfully synthesize a nanocrystalline cemented carbides with both high hardness and high fracture toughness. Our work is another example to prove that increasing the density of boundaries with high separation energy is a promising way to obtain material with both high hardness (or strength) and high fracture toughness. We can look forward to obtain many other excellent structural materials by following the same idea.

In the present works, the effect of coherency WC/Co phase boundaries on the fracture toughness of the nanocrystalline WC-Co cemented carbides is studied by MD simulation method. The simulation results confirm that the nanocrystalline cemented carbides with coherency WC/Co phase boundaries has higher fracture toughness than that without coherency WC/Co phase boundaries, which is in good agreement with our previous experimental works. Moreover, the mechanism of why coherency WC/Co phase boundaries can improve the fracture toughness of the nanocrystalline WC-Co cemented carbides has also been studied, it is found that the separation energy of the coherent WC/Co phase boundary is larger than those of incoherent WC/Co phase boundaries. Therefore, the coherent WC/Co phase boundary will effectively block the propagation of microcracks along them, and increases the probability of transcrystaline path through the FCC Co phase. The high ductility of the FCC Co phase can blunts the crack tip and increases the fracture toughness of the nanocrystalline cemented carbides.

## Methods

**Figure 7:**
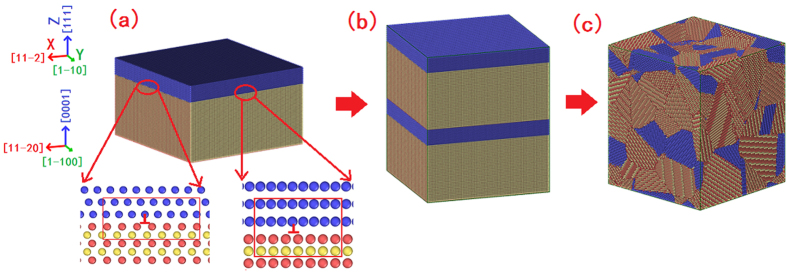
Simulation model of the nanocrystalline cemented carbide with coherent WC/Co phase boundary. Atoms colored by blue, red, and yellow represent Co, W and C atoms, respectively.

The construction of a nanocrystalline WC-Co cemented carbide model with coherent WC/Co phase boundaries can be divided into three steps. The first step is to construct a model with coherent WC/Co phase boundary (Fig. 7(a)). In order to using periodic boundary condition along the *x* and *y* directions, the minimum size of the model along the two directions must be 13.02 *nm* and 5.50 *nm*, respectively. In present work, the model sizes along the two directions are 26.04 *nm* (two times of 13.02 *nm*) and 27.50 *nm* (five times of 5.50 *nm*), respectively; the size along z direction is 17.13 *nm* (Fig. 7(a)). Because the lattice parameters of the two phases are not identical, the misfit dislocations can be created on the coherent WC/Co phase boundary (insert figure in Fig. 7(a)). In the present model, the Co phase is 13.1 wt.% (20 atom%). Then the model is doubled along *z* direction, and the model size along the x, y and z directions are 26.04 *nm*, 27.50 *nm* and 34.26 *nm*, respectively (Fig. 7b). Finally, Voronoi construction is applied to build the polycrystalline system. 3 × 3 × 4 = 36 “seeds” are randomly distributed along the x, y and z directions, respectively. Grains with random misorientations are generated from the “seeds” and filled the box with about 2.37 million atoms. Using this method, a nanocrystalline WC-Co cemented carbide model with coherent WC/Co phase boundaries (named as coherent model) is constructed (Fig. 7c). Furthermore, a same size nanocrystalline WC-Co cemented carbide model without coherent WC/Co phase boundary (named as incoherent model) is also constructed by using the similar method. The structure of the two models is the same except that one has coherent WC/Co phase boundaries and the other hasn’t, so the different mechanical properties of them must come from the joint types of the WC/Co phase boundaries.

## Additional Information

**How to cite this article**: Xie, H. *et al*. Effect of WC/Co coherency phase boundaries on Fracture toughness of the nanocrystalline cemented carbides. *Sci. Rep.* **6**, 31047; doi: 10.1038/srep31047 (2016).
